# Loss of toll-like receptor 4 ameliorates cardiovascular dysfunction in aged mice

**DOI:** 10.1186/s12979-021-00251-y

**Published:** 2021-11-05

**Authors:** Huan Liu, Shujuan Chu, Zhilin Wu

**Affiliations:** Anesthesiology Department, Union Hospital, Tongji Medical College, Huazhong University of Science and Technology, Wuhan, 430022 People’s Republic of China

## Abstract

**Background:**

Toll-like receptor 4 (TLR4) is a pattern recognition receptor of the innate immune system. TLR4 contributes to many aging-related chronic diseases. However, whether TLR4 is involved in cardiovascular injury during the aging process has not been investigated.

**Methods:**

The effects of TLR4 on the cardiovascular system of aged mice were investigated in TLR4^−/−^ mice. An intraperitoneal glucose tolerance test (IPGTT) and insulin sensitivity test (IST) were conducted to evaluate global insulin sensitivity. Echocardiography was used to measure cardiac structure and performance. An isolated artery ring assay was used to measure the vasodilator function of the thoracic aorta. The inflammatory response was reflected by the serum concentration of cytokines.

**Results:**

TLR4 expression increased in the hearts and aortas of mice in an age-dependent manner. Loss of TLR4 increased insulin sensitivity in aged mice. Moreover, loss of TLR4 improved cardiac performance and endothelium-dependent vascular relaxation in aged mice. Importantly, the increases in serum inflammatory cytokines and oxidative stress in the heart and aorta were also inhibited by TLR4 deficiency.

**Conclusion:**

In summary, loss of TLR4 improved cardiac performance and endothelium-dependent vascular relaxation in aged mice. The reduced inflammatory responses and oxidative stress may be the reason for the protective effects of TLR4 deficiency during aging. Our study indicates that targeting TLR4 is a potential therapeutic strategy for preventing aging-related cardiovascular disease.

## Introduction

With the improvement of health care, the lifespan of humans has been extended, and the elderly population is increasing globally [1]. Aging is one of the prominent risk factors for human chronic diseases, such as arteriosclerosis, diabetes, tumors, and heart failure [2–6]. The prevalence of cardiovascular diseases increases with age. Therefore, aging is an independent risk factor for cardiovascular diseases, which contribute to the increased morbidity and mortality in the aging population [7].

A growing amount of evidence suggests that inflammation plays a critical role in the physiological aging process. Many studies have shown that the activated chronic inflammatory response is involved in aging-related diseases. Aging-related inflammation is characterized by increased levels of IL-6, IL-1β, TNF-α and type I interferon. This chronic activation of the innate immune system in the absence of infection during the aging process is called inflammaging [8, 9]. The activated innate immune system in aging causes insulin resistance and oxidative processes, making the cardiovascular system more vulnerable to stress, thereby increasing the risk of cardiovascular diseases [10–12]. Moreover, some studies have shown that inhibiting inflammation could reduce the occurrence of cardiovascular diseases in aging [13, 14], suggesting the important role of inflammation in aging-induced cardiovascular injury.

The mechanism responsible for inflammaging is still far from clear. Metabolic disorders, mitochondrial dysfunction, DNA injury and autophagy deficiency are all involved in inflammaging. The damaged DNA or self-derived molecules released from damaged cells are called damage-associated pattern molecules (DAMPs). Usually, DAMPs are transferred into lysosomes and then degraded. The accumulation of excessive DAMPs will lead to inflammation. TLR4 is an innate immune receptor that specializes in sensing DAMPs [15]. When sensing DAMPs, TLR4 triggers intracellular signaling pathways in a MyD88-dependent and MyD88-independent manner, which subsequently activates downstream inflammatory responses, leading to the release of inflammatory factors [16]. Senescence and aging phenotype-related interferon regulator factor are also activated by the TLR4 pathway [17]. The TLR4 pathway has been reported to be related to chronic inflammation and the progression of a variety of diseases, such as diabetes, cardiovascular diseases, and even cancer [18–21]. More importantly, many studies have shown that the TLR4 pathway participates in inflammaging and neurodegeneration [22, 23]. In addition, inhibiting TLR4 protects against age-associated neurodegeneration [24]. However, whether TLR4 contributes to cardiovascular injury during the aging process has not been investigated.

The present study focused on exploring the relationship of TLR4 with cardiovascular injury during aging using TLR4^−/−^ aged mice and analyzing the effects of TLR4 knockout on insulin resistance, inflammation, and oxidative stress in aged mice.

## Methods

### Animals

All in vivo *studies* were approved by the Medical Ethics Committee of Tongji Medical College and performed according to the National Institutes of Health Guide for the Care and Use of Laboratory Animals. TLR4^−/−^ mice with a C57BL/6 J background were provided by GemPharmatech in the present study. Male mice were divided into the following groups according to age: Young group (3 months) and Aged group (24 months). Briefly, 4-week-old wild-type and TLR4^−/−^ mice were allocated to different cages, with 4–5 mice per cage. All mice were housed in a standard laboratory animal facility with a 12-h light and dark cycle to eliminate interference. The heart and aorta were harvested and stored at − 80 °C for subsequent measurements.

### Echocardiography

Echocardiology was used to detect cardiac performance. Mice were anaesthetized with 3% isoflurane for induction and 1.5% isoflurane for maintenance. Before echocardiography, the chest hair was removed, and then M-mode and 2-dimensional imaging were performed with a VisualSonics 2100 high-resolution imaging system. For analysis, all parameters were averaged over 5 cardiac cycles.

### Insulin sensitivity

An intraperitoneal glucose tolerance test (IPGTT) and insulin sensitivity test (IST) were conducted to evaluate global insulin sensitivity. After 8 h of fasting, mice were treated intraperitoneally with glucose (2 mg/kg) or insulin (0.5 U/kg). Then, the glucose concentration of tail venous blood was measured with a glucometer.

### Measurement of vasodilator function

Thoracic aortas were used for vasodilator function detection with a DMT620 Multi Myograph system. Mice were anaesthetized with 5% isoflurane, and the thoracic aortas were excised. After cleaning in PSS buffer, the aortas were cut into 3-mm segments. Krebs-Henseleit solution was heated to 37 °C and equilibrated with 95% O_2_ and 5% CO_2_ for 30 min. Two stainless-steel wires were passed through the ring of the aorta. Then, the aortic rings were mounted in a tunnel with passive tension. After 60 min of equilibration, the aortic segments were stimulated with KCl to test contraction activity. Only the aortic segment responding to KCl was used for the next step. Finally, the cumulative dose-dependent curve in response to acetylcholine (Ach) or sodium-nitroprusside (SNA) at different concentrations was recorded.

### Serum measurement

The concentrations of serum insulin, total cholesterol, and LDL cholesterol and the levels of IL-1β, TNF-α, IL-6 and IFN-β in serum were measured using commercial kits following the manufacturer’s instructions. All kits used were purchased from Shanghai Enzyme-linked biotechnology Co., Ltd.

### Western blotting

Protein was extracted from aortas of hearts using RIPA lysis with PMSF. Then, the tissue was homogenized, and tissue lysates were centrifuged for 15 min at 12000 g with a 4 °C centrifuge. After boiling with loading buffer, protein samples were separated by SDS–PAGE gels and transferred to a PVDF membrane. The PVDF membranes were probed with primary antibodies at 4 °C overnight. Primary antibodies against TLR4, p16, p53, SOD2, and GAPDH were purchased from Cell Signaling Technology and diluted to a concentration of 1:1000 (GAPDH, 1:5000). After washing and incubation with anti-rabbit secondary antibody for 1 h at room temperature, the blots were visualized with an ECL detection kit in a Bio–Rad ChemiDocXRS system.

### Statistics

Data are presented as the mean ± SEM. For comparisons, one-way ANOVA was performed followed by Tukey’s multiple comparison test. All statistical tests were performed with GraphPad Prism software (Version 9). *P* values less than 0.05 were considered significant.

## Results

### Protein expression of TLR4 increased in the aged hearts and aortas of mice

**Fig. 1:**
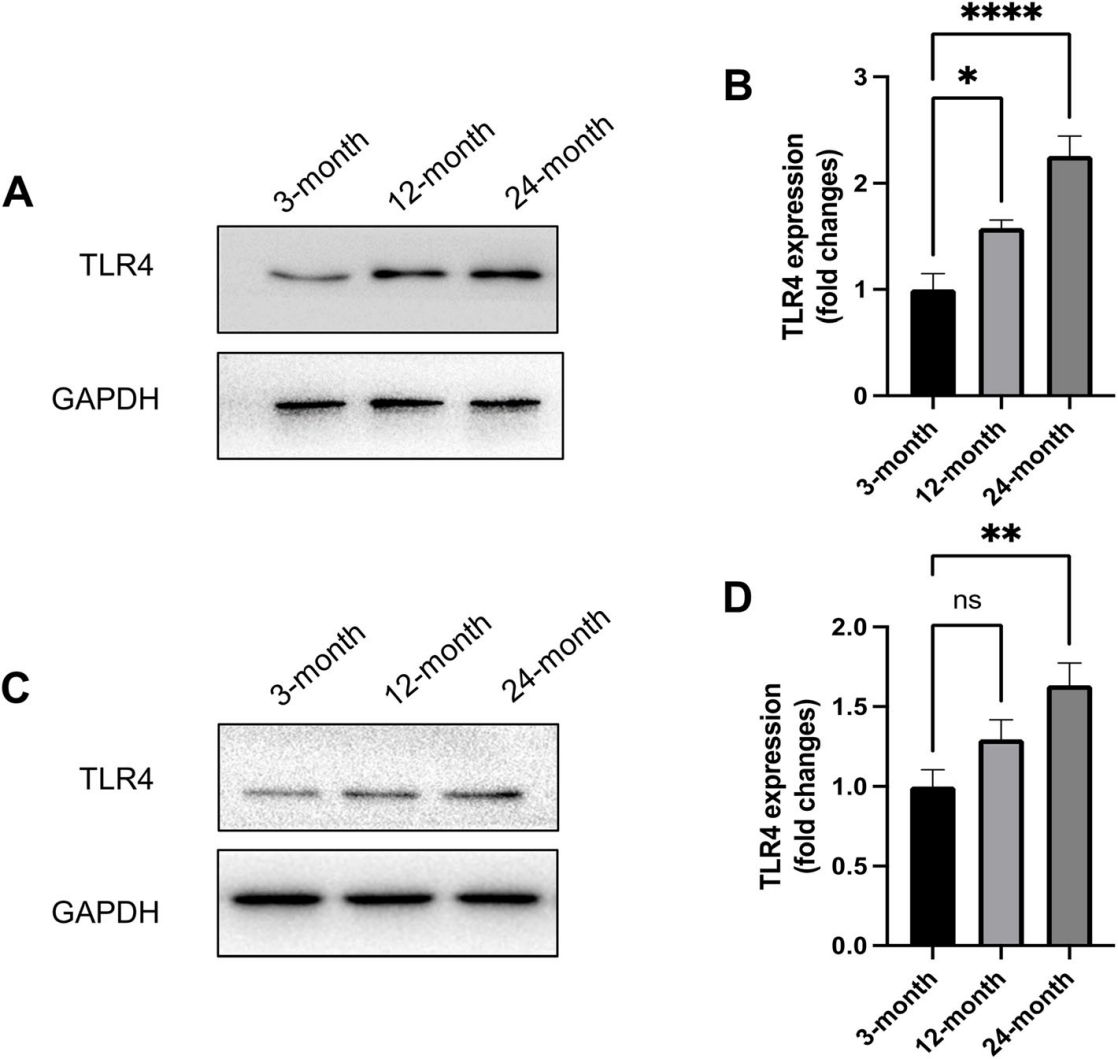
Protein expression of TLR4 increased in the aged hearts and aortas of mice. **A** Representative Western blots of TLR4 in the hearts of mice at different ages. **B** Statistical results of TLR4 expression in the heart. **C** Representative Western blots of TLR4 in the aortas of mice at different ages. **D** Statistical results of TLR4 expression in aortas. *n* = 6 for each group. ns, not significant. *P < 0.05, **P < 0.01, ****P < 0.0001

To determine whether TLR4 is related to cardiovascular dysfunction associated with aging, we measured the protein expression of TLR4 in the hearts and aortas of mice at different ages. The results indicated that TLR4 expression was significantly increased at 12 months and 24 months (Fig. 1A and B), suggesting an age-dependent increase in TLR4 expression in the heart during aging. Consistently, significantly increased TLR4 expression was also found in the aortas at 12 months and 24 months (Fig. 1C and D). All these results indicated that increased TLR4 plays roles in physiological cardiovascular aging.

### Effects of loss of TLR4 on insulin sensitivity and lipid profile in aged mice

**Fig. 2:**
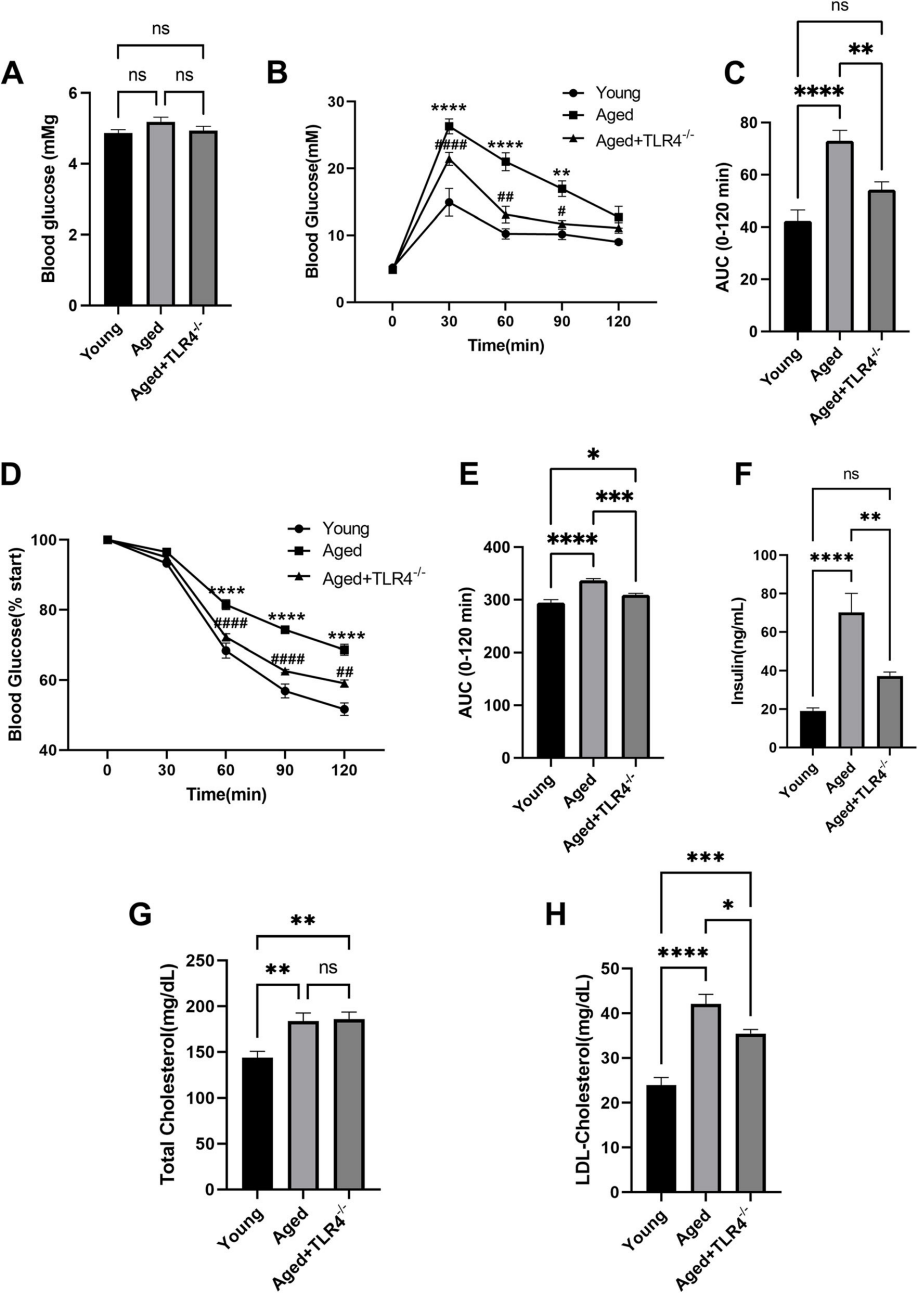
Effects of loss of TLR4 on insulin sensitivity and lipid profile in aged mice. **A** Fasting blood glucose in the mice of different groups. **B** and **C** Intraperitoneal glucose tolerance test (IPGTT) and insulin sensitivity test (IST) of the mice in different groups. **D**, **E** and **F** Serum insulin (**D**), total cholesterol (**E**), and LDL cholesterol (**F**) of the mice in different groups. *n* = 6 for each group. **P < 0.01, ****P < 0.0001 vs. Young Group. ^#^*P* < 0.05, ^##^*P* < 0.01, ^####^*P* < 0.0001 vs. Aged Group

Although fasting blood glucose was not different between the Young and Aged groups (Fig. 2A), the IPGTT (Fig. 2B and C) and IST (Fig. 2D and E) results indicated a significant reduction in whole-body insulin sensitivity. Moreover, loss of TLR4 effectivity improved insulin sensitivity in aged mice (Fig. 2B-E). The increase in serum insulin level was also attenuated by loss of TLR4 (Fig. 2F). Aging was related to increased total cholesterol and LDL cholesterol levels in serum (Fig. 2G and H). Although there was no significant effect on the total cholesterol level, loss of TLR4 significantly prevented the increase in LDL cholesterol (Fig. 2G and H).

### Effects of loss of TLR4 on cardiac function and senescence

**Fig. 3:**
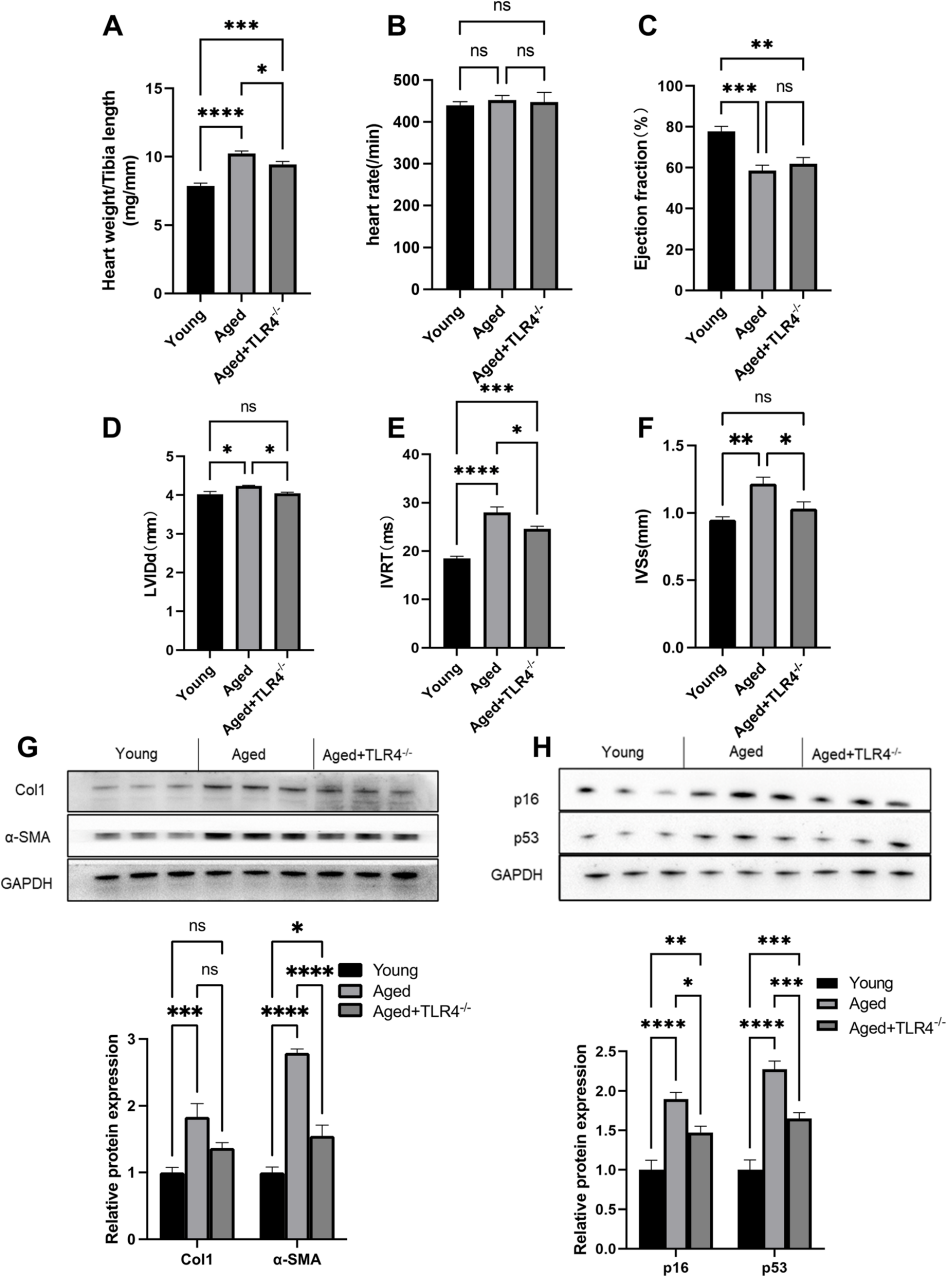
Effects of loss of TLR4 on cardiac function and senescence. **A** Heart weight/tibia length ratio of young, aged and TLR4^−/−^ aged mice. **B** Ejection fraction of mice in different groups. **C** and **D** Left ventricular end-diastolic diameter (LVIDd) and isovolumic relaxation time (IVRT) of mice in different groups. **E** Representative Western blots of p16 and p53 in the hearts of mice in different groups. **F** Statistical results of p16 and p53 in hearts. *n* = 6 for each group. ns, not significant. *P < 0.05, **P < 0.01, ***P < 0.001, ****P < 0.0001

Compared with the young group, the aged mice showed an increased ratio of heart weight/tibial length, suggesting cardiac hypertrophy (Fig. 3A). However, loss of TLR4 prevented aging-induced cardiac hypertrophy (Fig. 3A). The echocardiography assessment showed no difference in heart rate between groups (Fig. 3B). The ejection fraction was significantly decreased in aged mice, and loss of TLR4 showed no effects on the ejection fraction of aged mice (Fig. 3C). Aged mice showed a significant elevation in LVIDd (left ventricular end-diastolic diameter), IVRT (isovolumic relaxation time) and IVSs (end-systolic interventricular septal thickness); however, loss of TLR4 blunted the increases in LVIDd, IVRT and IVSs in aged mice (Fig. 3D-F). We also found that loss of TLR4 reduced α-SMA and collagen I protein expression in aged hearts (Fig. 3G). In addition, the age-dependent elevation of the cellular senescence markers p16 and p53 was also blunted by the loss of TLR4 (Fig. 3H).

### Effects of loss of TLR4 on vasodilatation and vascular senescence

**Fig. 4:**
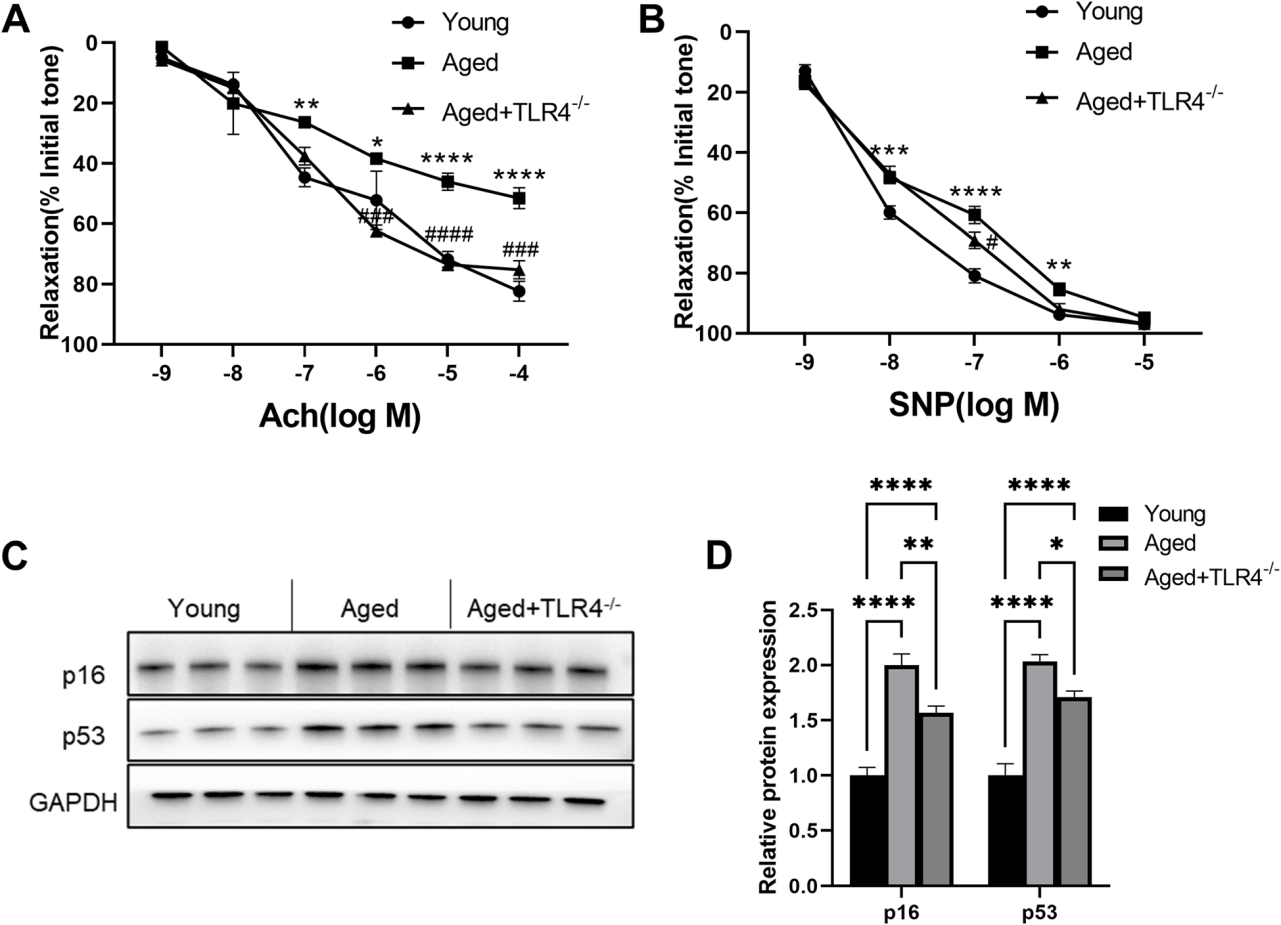
Effects of loss of TLR4 on vasodilatation and vascular senescence. **A** Effect of aging and TLR4 knockout on the endothelium-dependent vascular relaxation response to acetylcholine. **B** Effect of aging and TLR4 knockout on the endothelium-independent vascular relaxation response to sodium nitroprusside. **C** Representative Western blots of p16 and p53 in aortas of mice in different groups. **F** Statistical results of p16 and p53 in aortas. *n* = 6 for each group. *P < 0.05, **P < 0.01, ***P < 0.001, ****P < 0.0001 vs. Young Group. ^#^*P* < 0.05, ^###^*P* < 0.001 vs. Aged Group

As shown in Fig. 4A, the endothelium-dependent vascular relaxation induced by Ach was markedly decreased in the aorta of aged mice. However, this effect was almost restored by loss of TLR4 (Fig. 4A). Moreover, the endothelium-independent vascular relaxation response to SNP in the aorta of aged mice was only improved by loss of TLR4 at concentrations of 10^− 7^ (Fig. 4B). Similar to the results in the heart, the age-dependent elevation of p16 and p53 expression was also blunted in the aorta by loss of TLR4 (Fig. 4C and D).

### Effects of loss of TLR4 on serum inflammatory responses

**Fig. 5:**
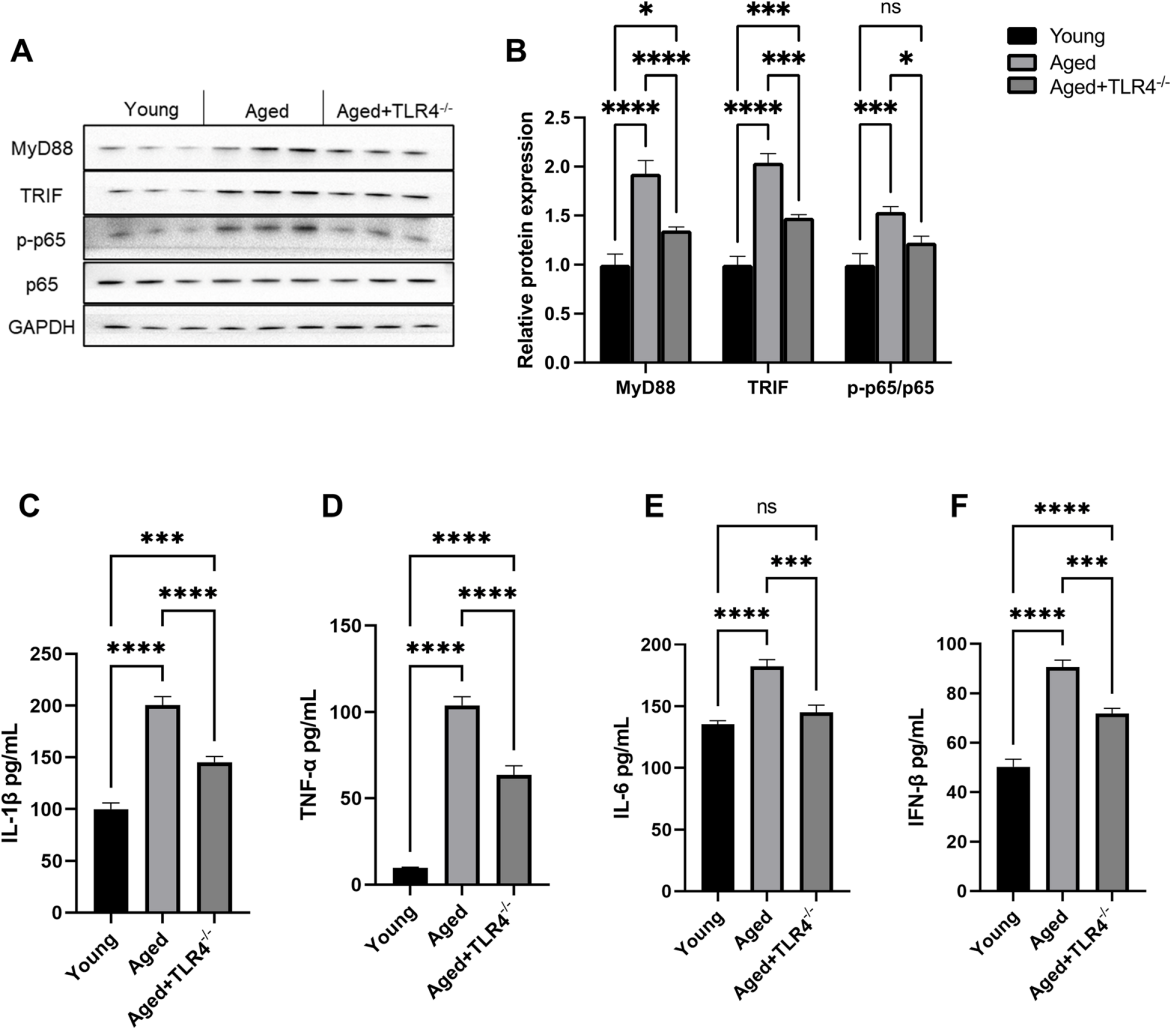
Effects of loss of TLR4 on serum inflammatory cytokines. **A-D** Serum concentrations of IFN-β, IL-1β, IL-6 and TNF-α in mice in different groups. ns, not significant. *P < 0.05, **P < 0.01, ***P < 0.001, ****P < 0.0001

TLR4 activates downstream signaling pathways and governs inflammatory responses via MyD88-dependent and MyD88-independent pathways. Our results showed that loss of TLR4 attenuated the elevated protein expression of MyD88 and TRIF and the phosphorylation of p65 in aged hearts (Fig. 5A and B). Moreover, compared with young mice, aged mice showed significantly increased serum inflammatory cytokines, including IFN-β, IL-1β, Il-6 and TNF-α, and loss of TLR4 reduced the serum levels of inflammatory cytokines in aged mice (Fig. 5C-F).

### Effects of loss of TLR4 on oxidative stress in the heart and aorta

**Fig. 6:**
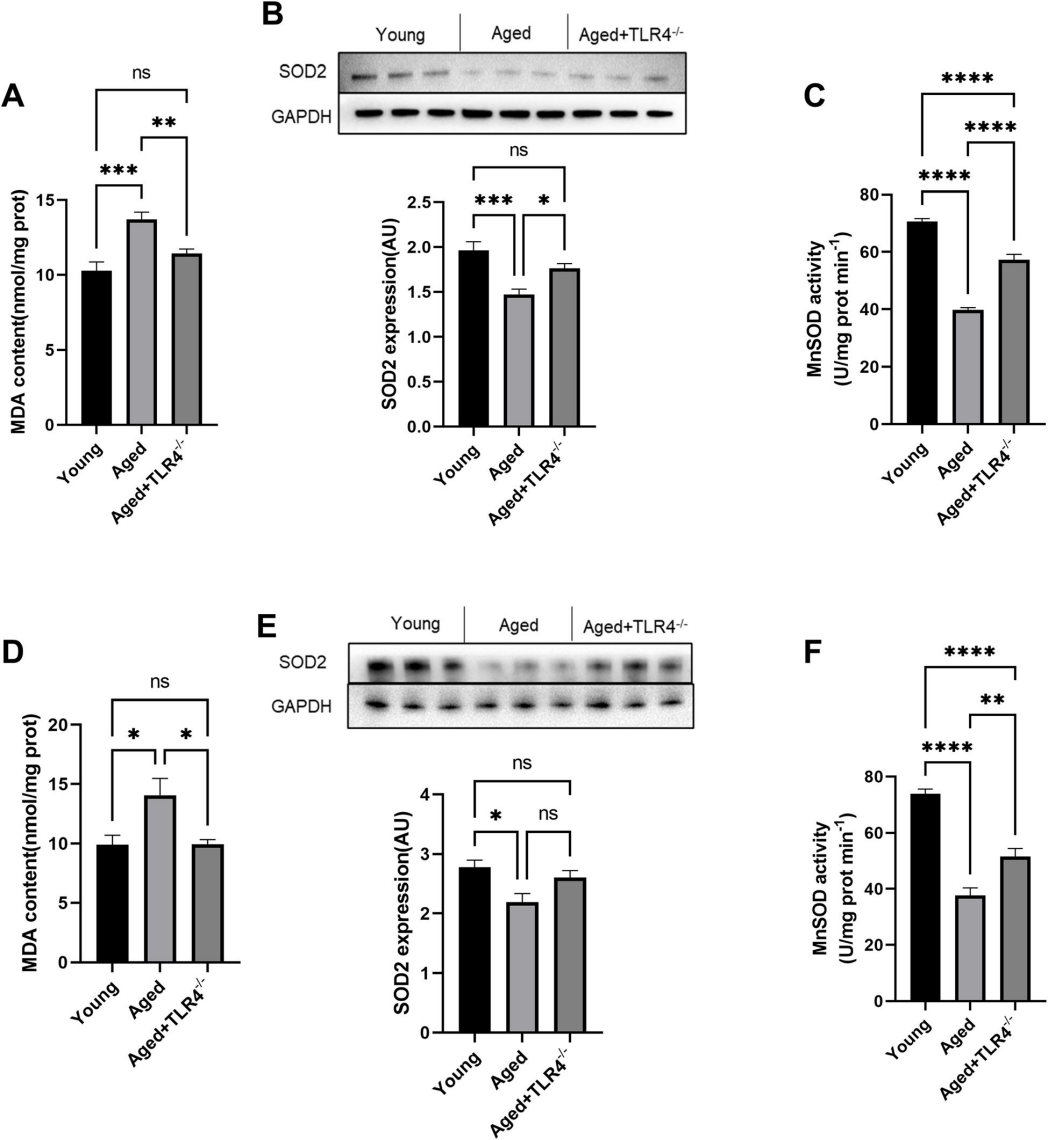
Effects of loss of TLR4 on oxidative stress in the heart and aorta. **A** MDA content in the hearts of different mice. **B** Representative Western blots and statistical results of SOD2 in the hearts of mice in different groups. **C** Cardiac MnSOD activity in different groups. **D** MDA content in aortas of different mice. **B** Representative Western blots and statistical results of SOD2 in aortas of mice in different groups. **C** Aortic MnSOD activity in different groups. ns, not significant. *P < 0.05, **P < 0.01, ***P < 0.001, ****P < 0.0001

The aged mice showed increased MDA content in hearts, and loss of TLR4 blunted this effect (Fig. 6A). Both the expression of SOD2 and the activity of MnSOD were decreased in the hearts of aged mice; however, loss of TLR4 significantly increased SOD2 expression and MnSOD activity in aged hearts (Fig. 6B and C). Similar to the results in the heart, the increased MDA content and decreased SOD2 expression and MnSOD activity were also blunted by loss of TLR4 in the aorta of aged mice (Fig. 6D, E and F).

## Discussion

In this study, we clarified the critical role of TLR4 in aging-related cardiovascular injury. We found that TLR4 expression increased in an age-dependent manner in both the heart and aorta. Deficiency of TLR4 improved insulin sensitivity and prevented the development of cardiovascular dysfunction and senescence in aged mice. Moreover, we found that TLR4 knockout downregulated serum proinflammatory cytokine expression and reduced oxidative stress in the cardiovascular system in aged mice. To the best of our knowledge, this study is the first to report the role of TLR4 in age-induced cardiovascular dysfunction. In summary, these findings provide direct evidence that TLR4 plays crucial roles in the pathology of cardiovascular aging and that inhibiting TLR4 is a potential therapeutic target for age-related cardiovascular disease.

Many studies have shown that the accumulation of DAMPs is one of the important causes of aging-related damage [25]. TLR4 is a predominant DAMP sensor that has been proven to mediate inflammation in many cell types. Moreover, some studies have reported that increased accumulation of amyloid β peptide and other DAMPs induces aging-associated inflammation and cellular senescence via the TLR4 pathway [26, 27]. Therefore, we hypothesized that TLR4 may be involved in the pathology of cardiovascular aging injury. Here, we found that TLR4 expression increased at 12 months and further increased at 24 months. These data strongly imply that TLR4 plays roles in the cardiovascular system during the process of aging. Then, we aimed to determine the effects of TLR4 on the cardiovascular system during aging.

Aging is associated with decreased insulin sensitivity and increased circulating cholesterol, which participates in the pathological process of cardiovascular dysfunction associated with aging [28, 29]. We investigated the effects of TLR4 on insulin sensitivity. The results showed that insulin sensitivity was significantly improved in TLR4^−/−^ aged mice, suggesting that TLR4 impairs insulin sensitivity during aging. Regarding circulating cholesterol, we found that loss of TLR4 reduced LDL cholesterol levels in aged mice but had no significant effect on total cholesterol. Given that LDL cholesterol is the main harmful lipid that accelerates vascular endothelial damage and atherosclerosis, these results suggested that TLR4 is important for age-related vascular injury. Together, these results indicated that loss of TLR4 mitigated metabolic disorders in aged mice.

In the process of aging, the cardiovascular system undergoes a series of pathophysiological changes, including cardiac hypertrophy, cardiac dysfunction, and impaired vasodilation. In this experiment, we revealed the important role of TLR4 in cardiac and vascular dysfunction during aging.

In aged mice, cardiac hypertrophy was evidenced by an increased ratio of heart weight/tibial length, and loss of TLR4 significantly prevented cardiac hypertrophy in aged mice. Moreover, impaired cardiac function was improved in TLR4^−/−^ aged mice, evidenced by decreased LVIDd and IVRT. Both endothelium-dependent and endothelium-independent vascular relaxation were impaired in aged mice. Remarkably, loss of TLR4 improved endothelium-dependent vascular relaxation, suggesting improved vascular function. Our results are consistent with a study in which inhibiting TLR4 alleviated pathological vascular injury during atherosclerosis.

p16 and p53 are age-dependent cellular senescence markers [30], and we found that increases in p16 and p53 expression was blunted by knocking out TLR4 in aged mice. Previous studies have also indicated that TLR4 promotes cellular senescence. Together with our findings, these results emphasize the important role of TLR4 in the pathological process of aging.

Inflammaging and increased oxidative stress are important manifestations of aging [31, 32]. We found that the expression of inflammatory cytokines in serum was significantly reduced in TLR4^−/−^ aged mice. These data indicated that loss of TLR4 prevents the process of inflammaging. We also found that loss of TLR4 attenuated the increase in protein expression of MyD88 and TRIF in aged hearts, suggesting that TLR4 could activate inflammation via both MyD88-dependent and MyD88-independent pathways during the aging process.

In both the heart and aorta, we found that loss of TLR4 reduced the MDA content and increased SOD2 expression and MnSOD activity, suggesting that loss of TLR4 reduced oxidative stress damage in the cardiovascular system of aged mice. Recently, many studies have shown that abnormal DNA damage induced by aging can activate inflammation via TLR4 and that TLR4 is crucial for the development of inflammation associated with cardiovascular disease, including cardiac remodeling, myocardial ischemia, and atherosclerosis [33–35]. In particular, a clinical study showed that TLR4 is associated with cardiac dysfunction in patients undergoing coronary artery bypass surgery [36], suggesting the relationship of TLR4 with human cardiovascular diseases. Our results demonstrated that TLR4 also participates in the process of cardiovascular dysfunction during the aging process, which further highlights the important role of TLR4 in the cardiovascular system. However, how TLR4 activation is induced in the cardiovascular system of aged mice still requires further investigation.

Despite the significance of those results, this study still has several limitations. First, our results showed the protective effects of TLR4 deficiency on aging-related cardiovascular dysfunction, and the underlying mechanisms may be related to the downregulated inflammatory response and oxidative stress. However, we cannot define the role of inflammation by regulating the levels of inflammatory factors. Second, we knocked out TLR4 from birth, and the effects of knocking out TLR4 in a later life stage were not investigated. Therefore, we cannot determine whether inhibiting TLR4 could reverse aging-related cardiovascular dysfunction. This concept needs to be addressed by inhibiting TLR4 in aged mice in further investigations.

## Conclusions

**Fig. 7:**
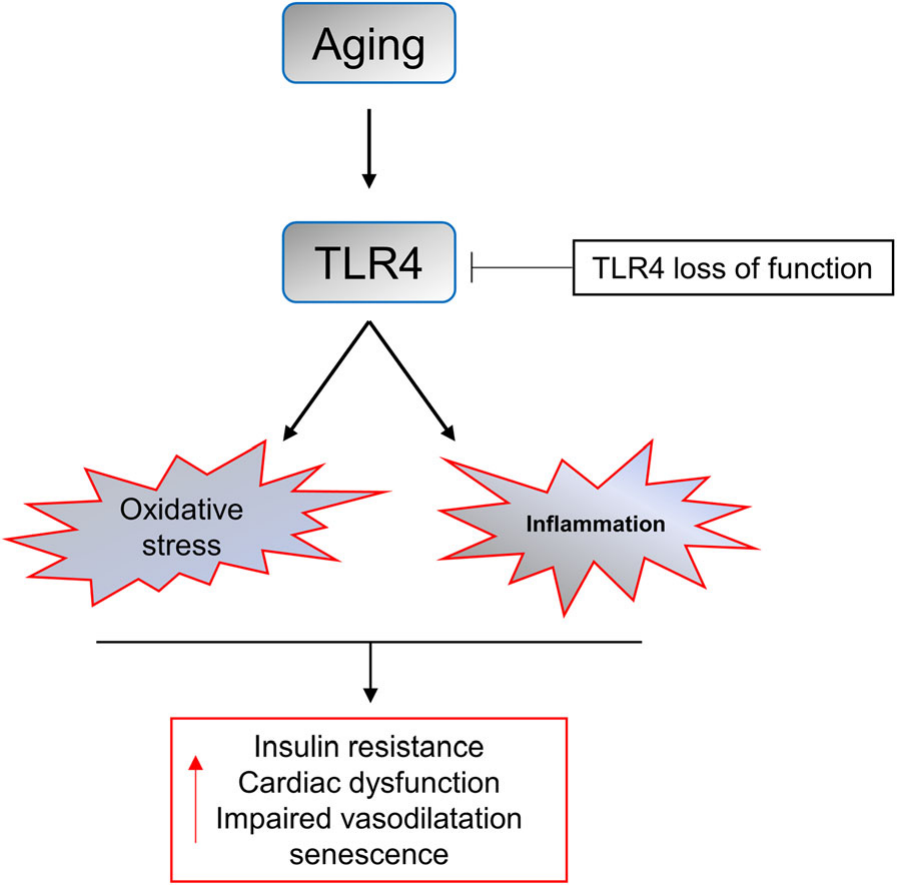
Model of TLR4-medicated cardiovascular injury during aging. TLR4-mediated signaling activation promotes inflammatory responses and oxidative stress in aged mice. Loss of TLR4 prevented cardiac hypertrophy and improved cardiac function and endothelium-dependent vascular relaxation in aged mice

In conclusion, this study demonstrated for the first time that TLR4 plays a crucial pathogenetic role in aging-related cardiovascular dysfunction (Fig. 7). Loss of TLR4 prevented cardiac hypertrophy and improved cardiac function and endothelium-dependent vascular relaxation in aged mice. The reduced inflammatory responses and oxidative stress may be the reason for the protective effects of TLR4 deficiency during aging. In summary, our study indicates that targeting TLR4 is a potential therapeutic strategy for preventing aging-related cardiovascular disease.

## Data Availability

All data are available on request from the corresponding author.
